# Osteoinductive 3D printed scaffold healed 5 cm segmental bone defects in the ovine metatarsus

**DOI:** 10.1038/s41598-021-86210-5

**Published:** 2021-03-23

**Authors:** Yunzhi Peter Yang, Kevin M. Labus, Benjamin C. Gadomski, Arnaud Bruyas, Jeremiah Easley, Brad Nelson, Ross H. Palmer, Kirk McGilvray, Daniel Regan, Christian M. Puttlitz, Alexander Stahl, Elaine Lui, Jiannan Li, Seyedsina Moeinzadeh, Sungwoo Kim, William Maloney, Michael J. Gardner

**Affiliations:** 1 Department of Orthopedic Surgery, School of Medicine, Stanford University, 240 Pasteur Drive, BMI 258, Stanford, CA 94304 USA; 2 Department of Material Science and Engineering, Stanford University, Stanford, USA; 3 Department of Bioengineering, Stanford University, Stanford, USA; 4 Department of Mechanical Engineering and School of Biomedical Engineering, Colorado State University, Fort Collins, USA; 5 Department of Clinical Sciences, Colorado State University, Fort Collins, USA; 6 Department of Microbiology, Immunology, and Pathology, Colorado State University, Fort Collins, USA; 7 Department of Chemistry, Stanford University, Stanford, USA

**Keywords:** Biomaterials - vaccines, Implants, Preclinical research, Translational research

## Abstract

Autologous bone grafts are considered the gold standard grafting material for the treatment of nonunion, but in very large bone defects, traditional autograft alone is insufficient to induce repair. Recombinant human bone morphogenetic protein 2 (rhBMP-2) can stimulate bone regeneration and enhance the healing efficacy of bone grafts. The delivery of rhBMP-2 may even enable engineered synthetic scaffolds to be used in place of autologous bone grafts for the treatment of critical size defects, eliminating risks associated with autologous tissue harvest. We here demonstrate that an osteoinductive scaffold, fabricated by combining a 3D printed rigid polymer/ceramic composite scaffold with an rhBMP-2-eluting collagen sponge can treat extremely large-scale segmental defects in a pilot feasibility study using a new sheep metatarsus fracture model stabilized with an intramedullary nail. Bone regeneration after 24 weeks was evaluated by micro-computed tomography, mechanical testing, and histological characterization. Load-bearing cortical bridging was achieved in all animals, with increased bone volume observed in sheep that received osteoinductive scaffolds compared to sheep that received an rhBMP-2-eluting collagen sponge alone.

## Introduction

Bone grafts are often required to reconstruct segmental bone defects caused by trauma or disease^1,2^. Autologous bone grafts generally demonstrate the greatest regenerative potential for tissue repair but are hampered by limited availability and significant complications at the donor site. Alternative grafting materials include allogeneic bone and synthetic void fillers, but these grafts display reduced healing efficacy. In very large bone defects greater than 4–5 cm, even traditional autograft is often insufficient to induce repair on its own. More complex reconstructive procedures are typically required. Current treatment options include distraction osteogenesis, induced membrane, and vascularized fibular transplantation^3^. Distraction osteogenesis uses bone tissue’s natural ability to lengthen under tension in order to generate new bone by slowly pulling apart broken ends^4,5^. The induced membrane technique is a two stage process involving implantation of a polymer spacer, around which a fibrovascular tissue envelope forms as part of the foreign body reaction. Later, the polymer spacer is removed, and the resulting cavity left within the tissue envelope provides a highly privileged environment for autograft regeneration^6,7^. Vascularized fibular grafting, which is a surgical procedure that transplants an intact cortical flap, complete with its vascular networks, to reconstruct the defect. Surgical anastomosis of minute blood vessels enables reestablishment of blood flow across the graft immediately following implantation, greatly improving the rates of graft success^8,9^. Each of the above clinical therapies is accompanied by significant drawbacks. Complications are frequent, and can lead to long-term patient disability, discomfort, and even amputation. Tissue engineered bone grafts are a promising area of research that might one day overcome the limitations of current bone grafting procedures by developing artificial constructs with improved potential to regenerate living bone. One promising approach is to combine a vascular pedicle with an advanced 3D printed scaffold to produce a prevascularized bone scaffold for single-phase surgical treatment^10,11^. Using the patient’s body as a bioreactor is an alternative strategy to prevascularize bone grafts for reconstruction of large bone defects^12,13^.

We previously developed 3D printed biodegradable scaffolds made from a composite of polycaprolactone (PCL) and β-tricalcium phosphate (β-TCP) for bone regrowth. To characterize the suitability of these constructs for use in bone grafting surgeries, we systematically studied their parameters and properties^14^ and the effect of terminal sterilization on scaffold mechanics and cytocompatibility^15^, as well as in vivo degradation^16^. Our 3D printed scaffolds have been used to aid union of critical size segmental bone defects in rats^17^ and to promote bone regeneration within large channels in the rabbit femoral head, both with and without osteonecrosis^18,19^.

Though PCL/β-TCP scaffolds provide structural support for the deposition of actively regenerating bone tissues, the ability of the scaffold material to promote new bone growth is limited. The application of growth factors to induce the regeneration of bone can work synergistically with synthetic scaffolds and has been used to increase the volume of bone growth on 3D printed scaffold structures for in vivo bone reconstruction^20–23^. Infuse (Medtronic) is a clinically approved collagen sponge-based delivery system for recombinant human bone morphogenetic protein-2 (rhBMP-2), which increases bone growth. However, the delicate spongy collagen structure is not stable under load-bearing conditions and surgical manipulation can induce unwanted factor release during implantation. Controlling the release of rhBMP-2 is critical for ensuring that the growth factor is present within therapeutic levels at the location of need during the healing process. Accidental release of large quantities of the growth factor during implantation risks impairing the regenerative potential of the implant and excessive BMP-2 release beyond recommended values has also been associated with adverse tissue responses such as bone resorption, cyst-like bone formation, and inflammation^24^. As such, we hypothesize that incorporation of the rhBMP-2 delivery system within a rigid PCL/β-TCP scaffold may yield an engineered bone graft with greater regenerative potential than either component alone.

**Figure 1:**
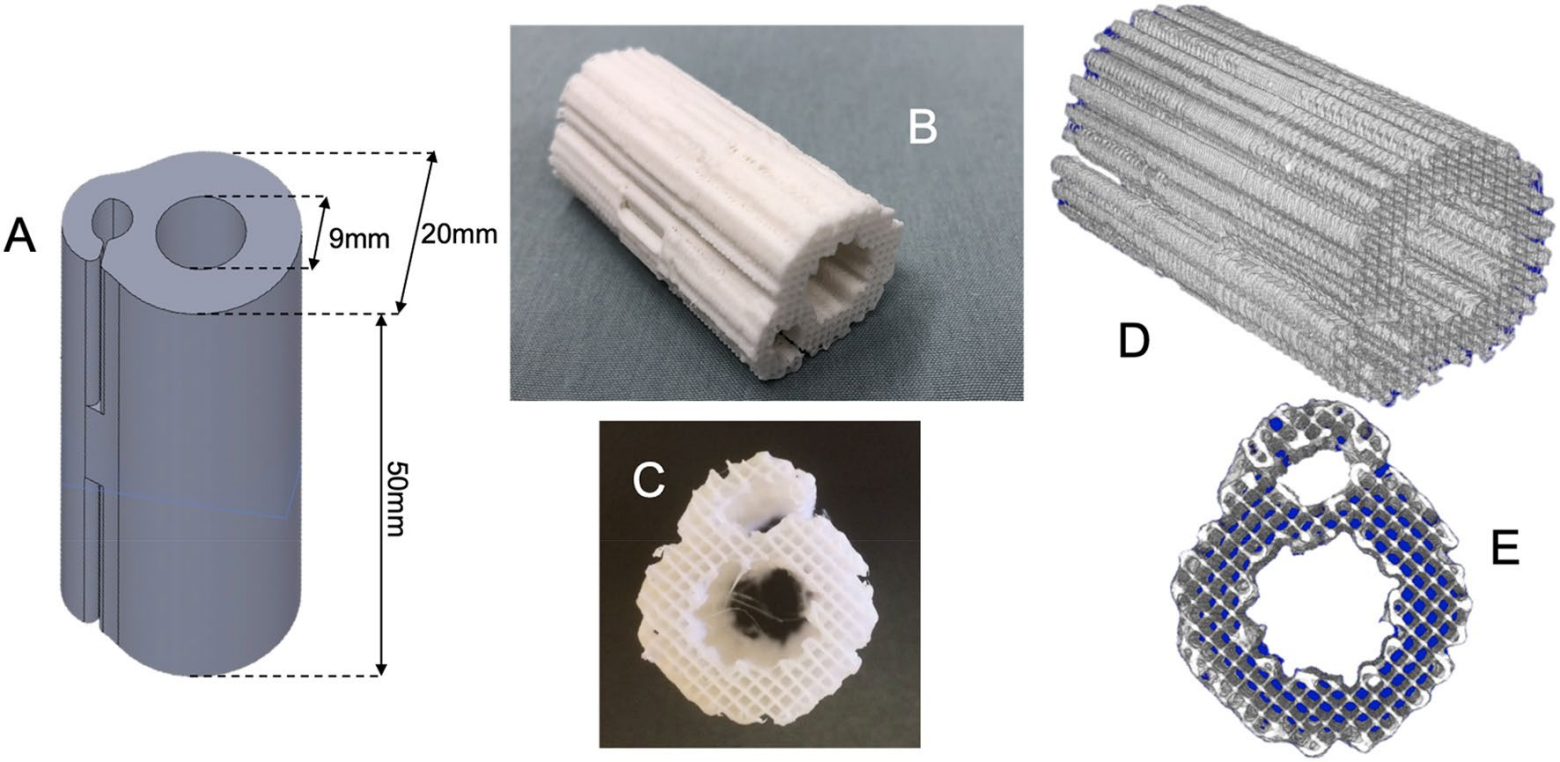
A 3D printed PCL/β-TCP scaffold with unique design. (**A**) Computer model of the scaffold’s geometry; (**B**,**C**) Photos of the scaffold after manufacturing; (**D**,**E**) micro-CT scaffold reconstruction (Scanco USA Inc., http://www.scanco.ch/).

In this study, we designed and 3D printed a novel PCL/β-TCP scaffold with a central channel for intramedullary nail fixation and a side hook to hold and stabilize Infuse rhBMP-2 collagen sponges (Fig. 1). There were two primary aims of this feasibility study. First, to investigate the effectiveness of the novel PCL/β-TCP scaffold loaded with rhBMP-2 to assist bone growth similarly to the gold-standard Infuse rhBMP-2 collagen sponge, and secondly, to determine animal tolerance to the use intramedullary nail fixation of a critically-sized defect using a normograde implantation approach. We tested the healing efficacy of this novel scaffold in a 5 cm critical size segmental bone defect in the ovine metatarsus. rhBMP-2 release by an Infuse sponge without a 3D printed scaffold was used as a control. In total, 4 sheep underwent ostectomy and treatment with rhBMP-2 with or without a PCL/β-TCP scaffold. After 24 weeks, quantification of new bone growth and mechanical characterization were performed to evaluate bone healing. Histological techniques were used to analyze the quality of the regenerated tissues.

## Results

### Scaffold fabrication and characterization

A novel PCL/β-TCP scaffold with a central channel for intramedullary nail fixation and side hooks to hold and stabilize an absorbable collagen sponge (Infuse bone graft) loaded with rhBMP-2 collagen sponges was designed as shown in Fig. 1A. Figure 1B,C show photos of a 3D printed PCL/β-TCP scaffold. Figure 1D,E show representative reconstructions of a 3D printed PCL/β-TCP scaffold obtained/captured by micro-computed tomography (micro-CT). As shown in Fig. 1B–E, the geometry of the device consists of a cylinder (outer diameter 20 mm, inner diameter 9 mm, length 50 mm; dimensions selected through consideration of the anatomy of the sheep metatarsus and clinically available IM nail size) with three consecutively aligned hooks, having alternating orientations, on the side. The central channel of the cylinder (Fig. 1C,E) is used to accommodate an IM nail for segmental bone defect stabilization/fixation. The hooks are used to contain an Infuse rhBMP-2 collagen sponge to induce bone formation (Fig. 1B,D). The hooks provide protection against sponge compression during and after implantation, which could lead to unwanted factor release. The pore size and porosity of the 3D printed scaffolds are approximately 820 μm and 65%, respectively, as measured with scanning electron microscopy (SEM) and 729 μm and 73% as measured with micro-CT.

### Biomechanical characterization

The mechanical properties of the healed metatarsus were examined by four-point bending and torsion testing. The four-point bending global stiffness was very similar between the scaffold and control treatment groups (Table 1). In the torsion tests, the scaffold treatment exhibited a slightly greater mean rigidity, ultimate torque, and yield torque compared to the control group (Table 1), although the small sample sizes of 2 sheep per group used in this study mean that further research is required to confirm this effect.

**Table 1:** Results from four-point bending and torsion tests, showing data from individual specimens and combined treatment means and standard deviations.

Treatment	Bending stiffness (N/mm)	Ultimate torque (N m)	Yield torque (N m)	Torsional rigidity (N m ^2^/radian)
Scaffold	780	24	16	12
Scaffold	1400	52	30	31
Mean ± standard deviation	1090 ± 440	38 ± 19	23 ± 10	21 ± 13
Control	1200	34	24	20
Control	950	15	12	11
Mean ± standard deviation	1070 ± 170	25 ± 14	18 ± 8	15 ± 7

### Micro-computed tomography

**Figure 2:**
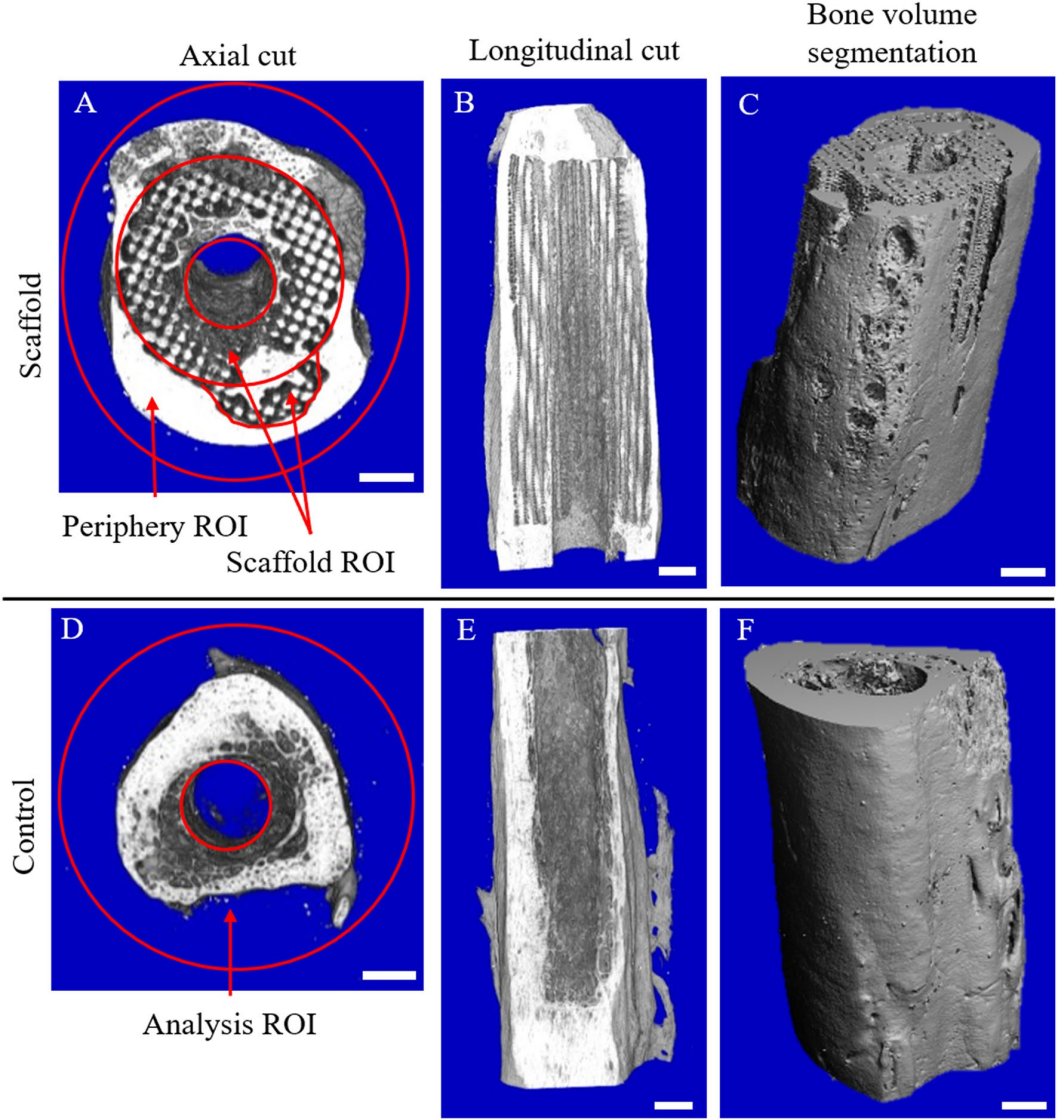
Micro-CT reconstructions images showing axial and longitudinal cut planes and obliques views of the bone volume segmentation for an example scaffold specimen (**A**–**C**) and control specimen (**D**–**F**). Micro-CT evaluations of bone and scaffold volume content were performed (**A**) within the 5 cm long scaffold ROI and around the periphery ROI of the scaffold specimens. The reported bone volume includes both the scaffold and periphery ROIs. For the control specimens, (**D**) the bone volume was analyzed in only a single region containing the entire defect region outside the intramedullary space. Scale bars indicate 5 mm. Images were generated by Scanco micro-CT 80 software (Scanco USA Inc., http://www.scanco.ch/).

Micro-computed tomography (micro-CT) was used to quantify the volume of bone tissue growth in the defect space. In the control specimens, the entire defect volume excluding the intramedullary space was examined as a single ROI. For the scaffold group, bone growth was measured in two regions of interest (ROIs): within the scaffold and on the scaffold periphery (Fig. 2). For comparison with the control group, the total bone volume was also analyzed to include both the scaffold and periphery ROIs. In all specimens, the volume of new bone was greater than that measured in an equivalent 5 cm segment of healthy metatarsus. For sheep that received scaffolds, the mean bone volume within the scaffold was about two times greater than in the peripheral ROI (Table 2). Within the scaffold ROI, the mean volume of bone was 9% greater than the mean volume of the scaffold, although the scaffold volume is likely overestimated due to overlap between the density of PCL/β-TCP and developing bone tissues. For comparison, approximately 1000 mm^3^ of bone was measured within the density threshold range of the scaffold in the control group, despite no scaffold existing (Table 2). Even with possible underestimation of bone volume in the scaffold group, the mean total bone volume was still 29% greater in the scaffold group compared to the control group (Table 2).

**Table 2:** Micro-CT outcome data for bone and scaffold volume.

Treatment	Bone volume in scaffold ROI (mm^3^)	Bone volume in periphery ROI (mm^3^)	Total bone volume (mm^3^)	Scaffold volume (mm^3^)	New bone volume/native bone volume
Scaffold	5920	1970	7890	5,530	1.52
Scaffold	5630	4370	10,000	5,090	1.93
Mean ± standard deviation	5780 ± 200	3170 ± 1690	8940 ± 1490	5310 ± 310	1.72 ± 0.29
Control	N/A	N/A	5420	900*	1.04
Control	N/A	N/A	8420	1060*	1.62
Mean ± standard deviation	N/A	N/A	6920 ± 2130	980 ± 120*	1.33 ± 0.41

Results from individual specimens are presented, as well as means and standard deviations. The scaffold and peripheral ROIs were not applicable for the control group.

*No scaffold was present in the control specimens. This scaffold volume represents an inherent error in using intensity thresholds to compute the volume.

### Histomorphometry

**Figure 3:**
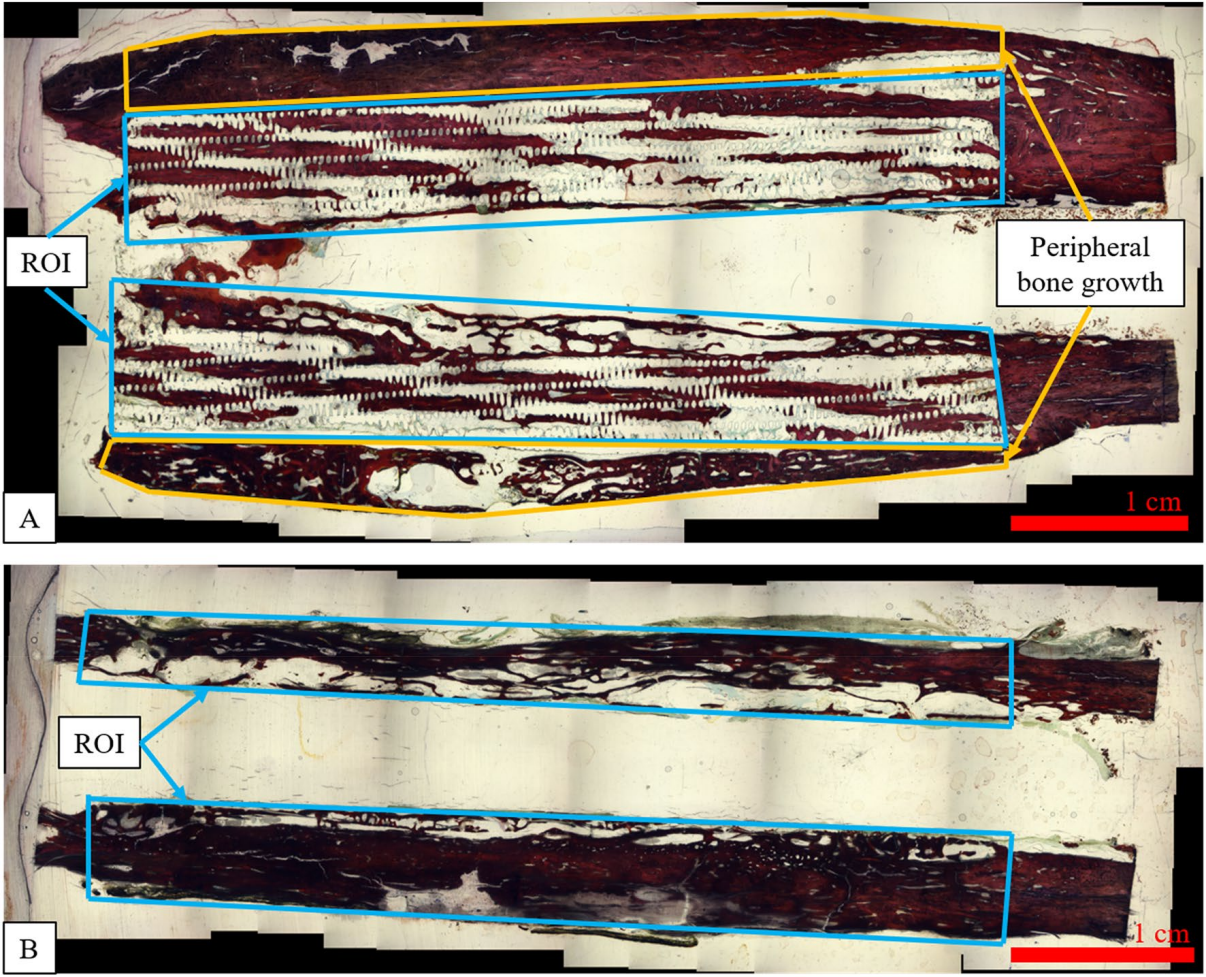
Histomorphometry evaluations of bone, scaffold, and soft tissue area fraction were performed within the ROI (bounded by blue). Example ROIs are shown for (**A**) the scaffold treatment group, in which the ROI is bounded by the scaffold perimeter, and (**B**) the control treatment group, in which the ROI is bounded by the new bone growth in the 5 cm defect region. Additionally, for the scaffold treatments, the area of peripheral bone growth was measured (bounded by yellow). Images were generated by ImagePro Premier software, version 9.3 (Media Cybernetics, https://www.mediacy.com/imageproplus).

Qualitatively, dense cortical bone was observed in the control group as well as in the peripheral region of the scaffold group, and there was bone growth throughout the pores of the scaffold (Fig. 3). Full histomorphometry results are shown in Table 3. Specimens treated with the Infuse sponge alone contained a greater area fraction of bone than those that received Infuse and the scaffold. This was due in part to the scaffold itself occupying about 20% of the measured area, which was not applicable to the control group. Additionally, the area fraction of soft tissue was greater in the scaffold group compared to the control group. On average, 50% of bone growth of scaffold specimens occurred around the scaffold periphery while 50% of bone growth was generated within the scaffolds.

**Table 3:** Histomorphometry results for the area fraction of each constituent in the scaffold and control treatment groups, and peripheral bone area in the scaffold group.

Treatment	Bone area fraction (%)	Scaffold area fraction (%)	Soft tissue area fraction (%)	Peripheral bone area (mm^2^)	Peripheral bone area/implant bone area (%)
Scaffold	39	17	23	63	14
Scaffold	38	21	18	316	86
Mean ± SD	39 ± 0.5	19 ± 3	20 ± 3	190 ± 180	50 ± 51
Control	79	N/A	6	N/A	N/A
Control	77	N/A	11	N/A	N/A
Mean ± SD	78 ± 1	N/A	9 ± 3	N/A	N/A

Results from individual specimens are presented, as well as means and standard deviations. The scaffold area fraction and peripheral bone area parameters were not applicable for the control group.

### Histopathology

**Figure 4:**
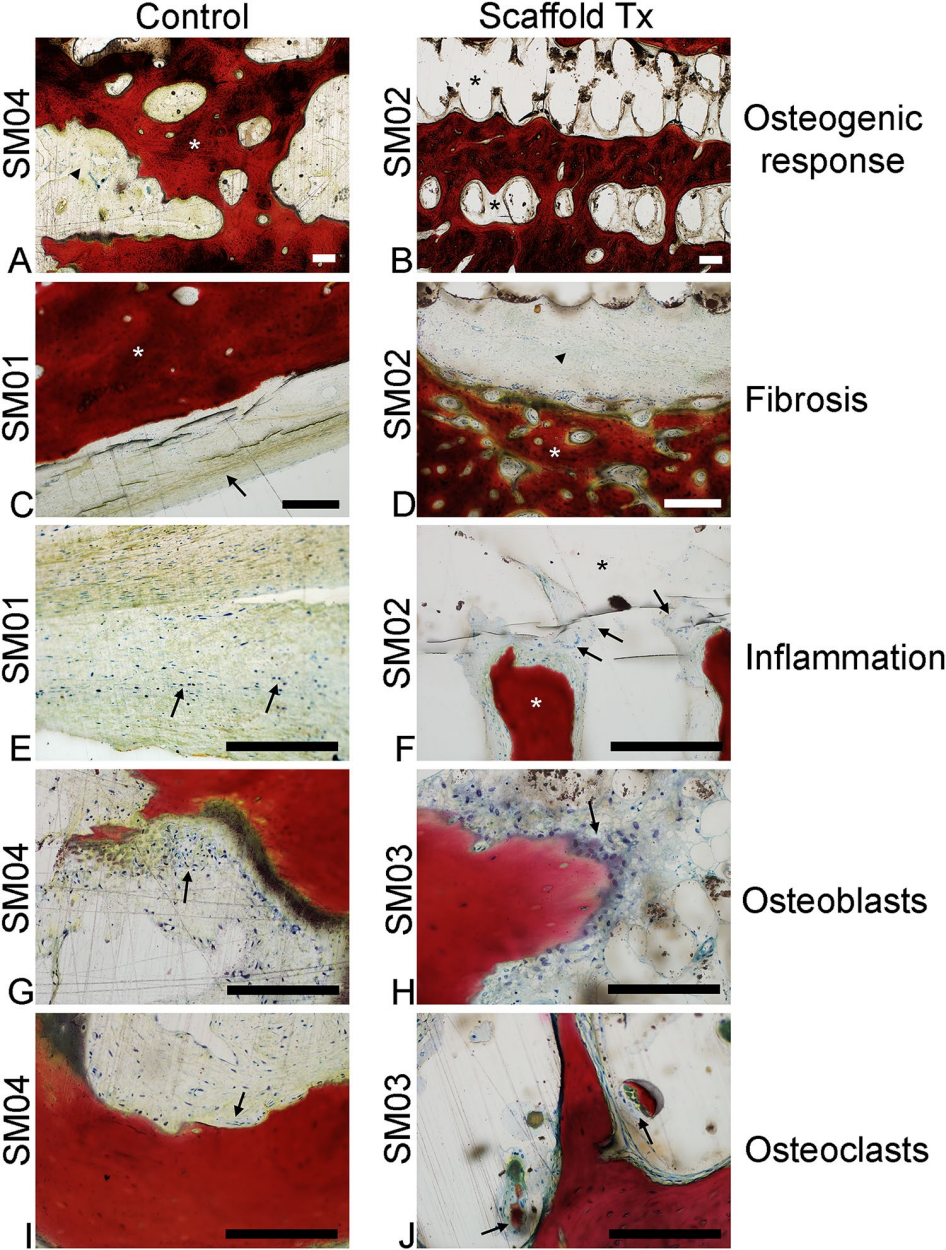
Representative photomicrographs of ISO-10993-6 Histological Scoring Parameters. (**A**,**B**) Overall osteogenic response (total new bone production) scores of 4 in both a control (SM04) and scaffold-treated animal (SM02). Tissue sections are predominated by anastomosing trabeculae of new bone (white asterisk). Intertrabecular spaces are composed of either fibrosis (arrowhead, **A**) or void space left from the implant (**B**). ×4 magnification. (**C**,**D**) Images demonstrating fibrosis scores of 1 in both a control (SM01) and scaffold-treated animal (SM02). Thin bands of dense fibrous connective tissue (arrow in **C**, arrowhead in **D**) are juxtaposed between new bone and defect or implant void spaces. ×10 magnification. (**E**,**F**) Image demonstrating cumulative inflammation scores of 2 in a control animal (SM01) and 5 in a scaffold-treated animal (SM02). Inflammation in control animals (**E**) was characterized by few scattered lymphocytes and plasma cells (arrows) within the fibrous tissue, while scaffold-treated animals (**F**) were characterized by infiltrates of macrophages and multi-nucleate giant cells (arrows) present in the fibrous tissue between new bone (white asterisk) and the implant void space (black asterisk). ×20 magnification. (**G**,**H**) Osteoblast activity scores of 2 in a control animal (SM04) and 1 in a scaffold-treated animal (SM03). Few clusters of plump osteoblasts (arrows) segmentally line endosteal surfaces of new bone. ×20 magnification. (**I**,**J**) Osteoclast activity scores of 1 in both a control (SM04) and scaffold-treated animal (SM03). Rare osteoclasts (arrows) were observed along endosteal surfaces of new bone. ×20 magnification. All scale bars indicate 200 μm. Images were captured with an Olympus BX51 microscope and Olympus DP73 camera using Olympus cellSens Entry software (version 2.3, https://www.olympus-lifescience.com/en/software/cellsens/).

The scaffold and control groups had similar osteogenic response scores (Table 4) when graded on a scale from 0 to 4, where 0 indicated no new bone tissue and 4 indicated the complete filling with new bone tissue (Fig. 4). Significant new bone growth was observed within and completely encapsulating the PCL/β-TCP scaffolds. Throughout the scaffold group, new bone production was characterized by a mixture of densely packed broad trabeculae and cortical bone containing prototypical osteon structures. Both the trabecular and cortical bone was comprised primarily of mature lamellar bone, with a lesser amount of immature woven bone component. In sections obtained from the control group, new bone formation resembled long bone cortices and was composed of mature lamellar bone arranged predominantly in a compact cortical architecture with minimal amounts of broad compressed trabecular bone adjacent to the intramedullary space. In both groups, minimal osteoblast and osteoclast activity was observed along endosteal surfaces of the new bone, consistent with the predominate presence of remodeled mature bone (Fig. 4).

**Table 4:** Histopathology scores for individual animals.

Treatment (Animal ID)	PMN	LC	PC	MF	GC	CIS	Nec	OB	OCr	NVas	Fib	ID	PD	OGr
Scaffold (SM02)	0	1	0	2	2	5	0	1	0	1	1	0	0	4
Scaffold (SM03)	0	1	0	2	2	5	0	1	1	1	2	0	0	3
Control (SM01)	0	1	0	1	0	2	0	1	1	1	1	0	0	3
Control (SM04)	0	1	0	1	0	2	0	2	1	1	2	0	0	4

Graded categories are polymorphonuclear cells (PMN), lymphocytes (LC), plasma cells (PC), macrophages (MF), giant cells (GC), cumulative inflammation score (CIS); the sum of scores assigned to PMN, LC, PC, MF, and GC categories; maximum possible score = 20), necrosis (Nec), osteoblast cells (OB), osteoclast remodeling (OCr), neovascularization (NVas), fibrosis (Fib), implant degradation (ID), paticulate debris (PD), and osteogenic response (OGr).

**Figure 5:**
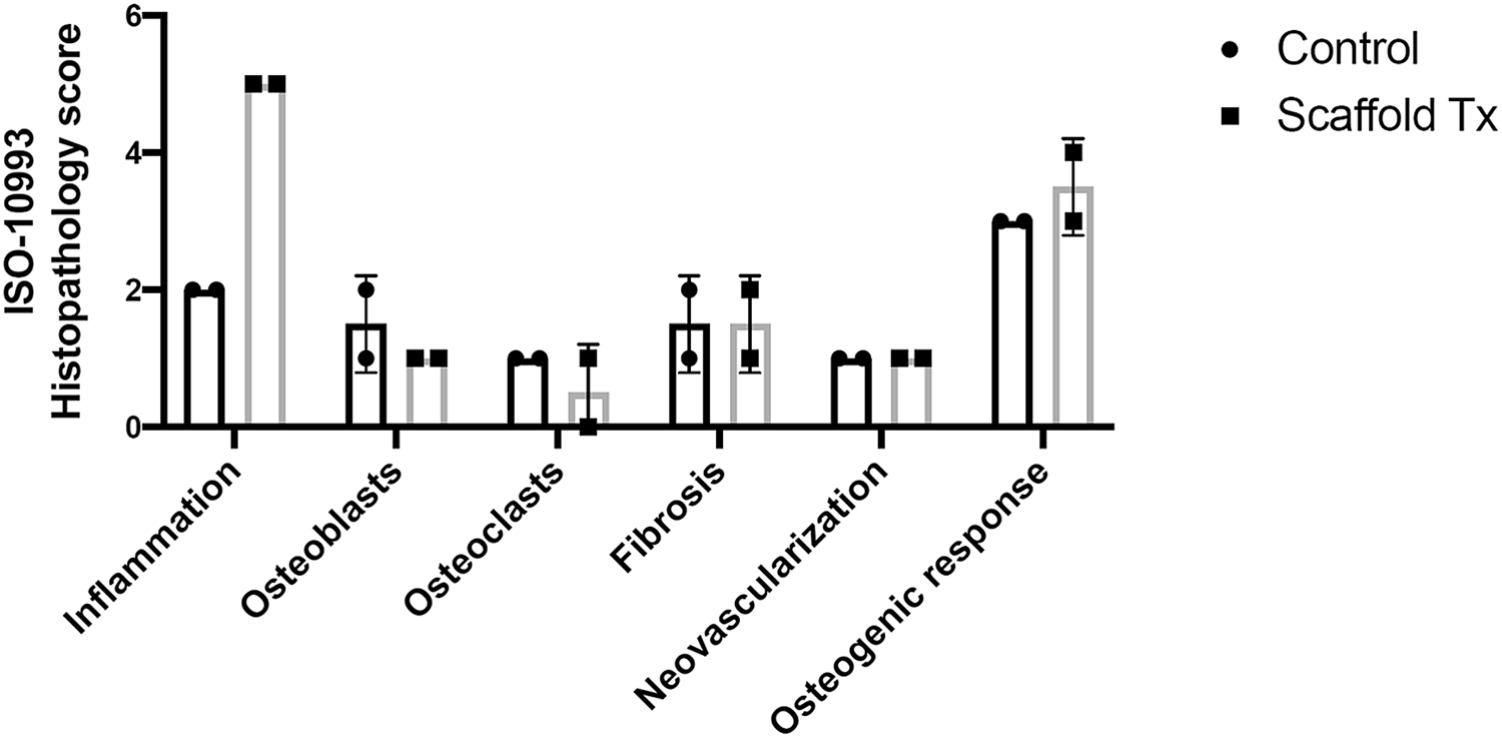
Mean histopathology scores of control and scaffold-treated groups for the selected histological features shown in Fig. 4. Figure was created with GraphPad Prism (version 9.0.1, https://www.graphpad.com/scientific-software/prism/).

The observed inflammatory responses also differed between the scaffold and control groups (Fig. 5). Scaffold animals had a greater degree of inflammation present in the fibrous tissue situated between the new bone and the implant void space. In the scaffold group, the inflammatory response was characterized by both a slightly greater density of inflammatory cells as well as different cell types compared with control animals (Fig. 4). The scaffold group predominantly contained multinucleated giant cells admixed with fewer macrophages and lymphocytes. In contrast, observed inflammation in control animals was minimal and consisted of only a few scattered lymphocytes present within the fibrous tissue.

No significant histological differences were observed in the degree of fibrosis or neovascularization between scaffold and control animals (Table 4). Additionally, no histological evidence of necrosis, implant degradation nor particulate debris was observed in any of the evaluated slides.

## Discussion

Delivery of BMP growth factors can increase the regenerative potential of bone grafts, enabling them to be used to heal larger defects than they otherwise could. In a 3 cm critical size segmental tibial bone defect study in sheep, 3D printed PCL/β-TCP scaffolds alone did not achieve bone union after 12 months, whereas bone bridging was observed within 3 months for a scaffold loaded with rhBMP-7. After 12 months, bone healing in the 3D printed PCL/β-TCP scaffolds loaded with rhBMP-7 exceeded even that of the group treated with autograft in terms of the volume of new bone and its mechanical properties^25^. A recent sheep pilot study also reported that a 3D printed calcium phosphate scaffold with an included vascular pedicle promoted greater bone formation than the scaffold alone at 3 months after implantation in a 3.5 cm critical size segmental metatarsal defect^10^. For the treatment of long bone nonunion, rhBMP-2 has yielded even better results than rhBMP-7 with regards to both overall rates of healing as well as the average time to union^26^.

In human patients, a randomized, controlled clinical investigation reported that rhBMP-2 delivery combined with allografts is safe and is as effective as traditional autologous bone grafts for the treatment of tibial fractures associated with extensive traumatic diaphyseal bone loss in defects ranging from 1 to 7 cm in length (mean = 4 cm)^27^. A second clinical analysis found that treatment with rhBMP-2 in combination with a calcium sulfate or calcium phosphate bone void filler achieved union in 16 out of 19 segmental bone defects ranging from 1.5 to 8.0 cm (mean = 4.75 cm)^28^.

Selecting an appropriate growth factor dosage is an important parameter in determining the outcome of reconstruction. In preclinical large animal models rhBMP-2 concentrations have been administered in the range of 0.10 to 0.80 mg/mL^29–32^. Dosages of 0.75 mg/mL and 1.5 mg/mL have been applied in humans^33^; and concentrations of approximately 0.4 mg/mL represent a typical dosage for sheep and dogs^31,32,34–36^. As such, we selected a dose of 0.4 mg/ml of rhBMP-2 for this sheep study.

In a canine study by Boyce et al., the healing of a 5 cm tibial defect was examined in three different treatment groups: rhBMP-2 combined with a collagen/ceramic matrix, rhBMP2 combined with autologous bone grafts, and autologous bone grafts alone^31^. In the dogs, the 5 cm defect constituted a loss of greater than 25% of the mean tibial length, which Boyce et al. suggest equates to roughly a 10 cm defect in the human tibia. In our study, the 5 cm defect constituted a loss of greater than 26% of the mean metatarsal length in the sheep. To our knowledge, this canine study and our ovine study represent the nearest comparison to the critical size bone defects in humans in terms of clinically relevant defect size and the proportional scale of defect size—larger than any other preclinical studies reported in the literature.

In such a substantial defect length, Boyce et al.found that none of the dogs treated with autograft alone had healed after 12 weeks. By contrast, dogs receiving rhBMP-2 in combination with autografts or the collagen ceramic matrix had healed by around 6 weeks, though the load bearing capabilities of the repaired tibiae were not evaluated. This study confirmed a well-known clinical observation that the outcome of using autografts (the gold standard grafting material) for such large bone defects becomes unreliable and reconstruction may require additional measures. However, as the authors acknowledged, a significant drawback of their study was that the periosteum remained intact after the osteotomy, whereas in clinical scenarios this is unlikely to be the case when massive bone loss occurs. Typically, clinical bone healing will be significantly impaired due to missing periosteum. As such, in the present study, we tested our approach in a clinically relevant critical size bone defect (5 cm) in the absence of periosteum. Successful bridging was observed in all sheep, demonstrating that rhBMP-2 delivery can indeed induce bone healing even in critical size segmental defects with compromised periosteum. To evaluate functional healing, the mechanics of the regenerated bone tissues were quantified. Similar bone mechanical properties were observed in both the scaffold and control groups, with torsional strength being perhaps slightly elevated in the scaffold group. Including an osteoconductive 3D printed scaffold significantly enhanced the overall volume of new bone tissue generated, with histological analysis confirming the presence of mature cortical bone tissue in both groups. Mild inflammation was noted within the PCL/β-TCP scaffold, as is typical of the foreign body reaction induced by implanted polymeric materials. Although small sample sizes in the study and the limitation of the two study groups preclude significant statistical analysis between the groups, this study was designed as a preliminary feasibility pilot study that sought to answer to main questions. First, to the best of our knowledge, this is the first study that has used intramedullary nailing as the primary fixation for a critically-sized bone defect in the sheep metatarsus. A major aim of this study was to determine animal tolerance to this procedure. The second question we sought to answer was whether this novel bone scaffold loaded with rhBMP-2 could achieve similar healing outcomes as what would typically be considered a positive control group, in this study that was a collagen sponge loaded with rhBMP-2. The results of this study suggest that the answer to both questions is yes, animals tolerate this surgical procedure very well and the bone scaffold with rhBMP-2 can produce similar healing outcomes to a collagen sponge with rhBMP-2. We are excited to disseminate these findings to the scientific community and anticipate following these results with a more rigorous, fully statistically powered study as subsequent work.

## Materials and methods

### Scaffold design

We designed and 3D printed a novel PCL/β-TCP scaffold with a central channel for intramedullary nail fixation and a side hook to hold and stabilize Infuse rhBMP-2 collagen sponges. The geometry of the implant consists of a general tubular structure, on the outer surface of which, flexible hooks were integrated, as shown in Fig. 1A, to create an additional longitudinal side channel. The inner diameter of the scaffold’s central channel was designed to fit a metal intramedullary rod of 9 mm in diameter used for implant fixation. The geometry of the implant was generated using Solidworks (Dassault Systèmes, Vélizy-Villacoublay, France).

### Material synthesis and scaffold manufacturing

PCL (M_n_ = 80,000 g/mol; Sigma-Aldrich, St. Louis, Missouri) and β-TCP powder with a mean particle size of 100 nm (Berkeley Advanced Materials Inc., Berkeley, California) were combined with a respective weight ratio of 80% and 20%. To this end, a solution of PCL dissolved in dimethylformamide (10% wt/v) and a suspension of β-TCP particles in dimethylformamide (5% wt/v) were prepared and stirred separately for 3 h at 80 °C. They were then mixed together and thoroughly stirred for another hour. This mixture was precipitated in excess water at 20 °C in order to remove the solvent before being dried at room temperature for 24 h under airflow. The resulting composite material was processed into 5 mm diameter pellets. The ratio of PCL to β-TCP in the pellets was validated using thermal gravimetric analysis, (Q500 TGA, TA instrument, New Castle, Delaware). Briefly, a sample of the composite material was heated to a temperature of 550 °C at a rate of 20 °C/min to burn off the organic polymer component. The remaining mass at the end of the test yielded the mass of the β-TCP portion present in the initial sample.

The solid pellets of PCL/β-TCP composite were then shaped into a filament for FDM additive manufacturing. Using a filament extruder, built in house, the pellets were heated to 90 °C and extruded at a constant speed to form a filament. The diameter of the filament was measured every 10 cm with calipers in order to ensure filament quality for printing reproducibility.

Prior to manufacturing, the scaffold Solidworks file was processed using a slicing software (Slic3r, version 1.2.9, www.slic3r.org) to generate G-code instructions to guide the 3D printer nozzle. An alternating layer-by-layer crisscross pattern was selected to obtain a scaffold with interconnected pores. Each layer consisted of parallel struts oriented orthogonally to the previous layer. Strut width was set to 350–400 µm, the distance between adjacent struts to 1.19 mm, and the height of each layer to 200 µms. The speed of the nozzle’s movement was set to 5 mm/s and its temperature to 160 °C. 14 scaffolds were manufactured using a Lulzbot Mini (Aleph Objects Inc., Loveland, Colorado) with a nozzle diameter of 500 µm. After manufacturing, the porosity of each scaffold was measured to assess fabrication reproducibility. 3D reconstructions of each sample were acquired using micro-CT imaging (eXplore CT120). They were then processed using MicroView Standard 2.5.0 (Parallax Innovations, Ilderton, Canada) in order to obtain the exact volume of the porous scaffolds.

The PCL/β-TCP 3D printed scaffolds were individually placed in a 5 M sodium hydroxide solution for 12 h at room temperature in order to increase scaffold surface hydrophilicity and nanoporosity for improved cell attachment. They were then rinsed 3 times in deionized water and dried overnight. For sterilization, the samples were first immersed for 20 min in a 70% ethanol solution, before being rinsed three times in PBS and dried overnight. They were then packaged in a sterile pouch and exposed to E-beam irradiation with a standard single dose of 25 kGy, following the guidelines in ISO 11137- 2:2006. The sterile scaffolds were stored in a dark environment at room temperature until implantation.

### Surgical procedure

**Figure 6:**
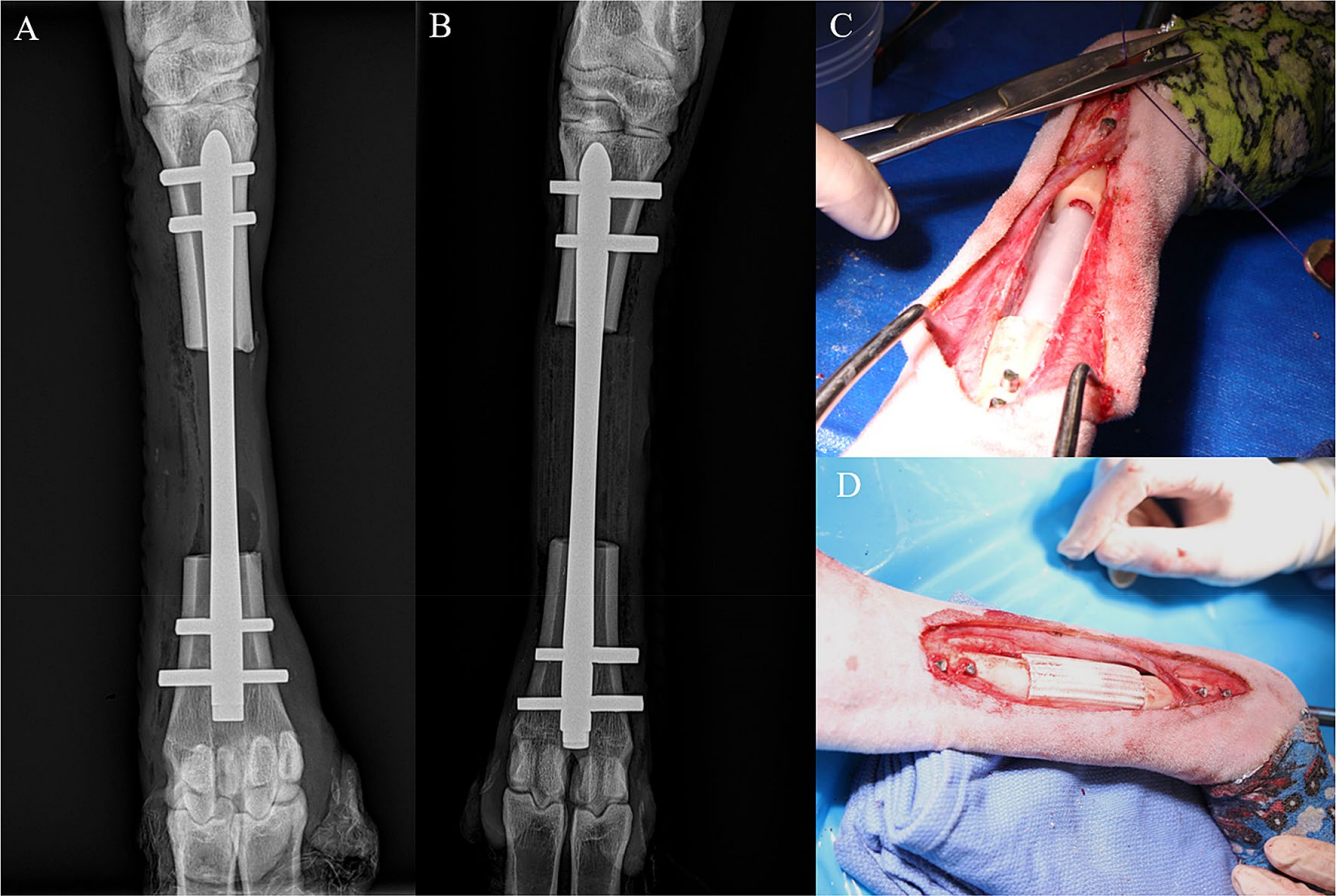
The surgical procedure included a 5 cm ostectomy repaired with an intramedullary nail. The ostectomy gap was filled with either the scaffold and a collagen sponge soaked with rhBMP-2, or a collagen sponge soaked with rhBMP-2 only (control group). Radiographs are shown at the time of surgery for the control (**A**) and scaffold (**B**) groups, and images of the open surgical site are shown for control (**C**) and scaffold (**D**) groups. Radiographs were taken using Smart DR system (Sound Imaging, Carlsbad, CA, https://soundvet.com/).

Colorado State University Institutional Animal Care and Use Committee (approval #A3572-01) approved the animal experiments and all experiments were performed in accordance with ARRIVE guidelines and regulations. Four female Columbia-Rambouillet sheep were used in this study. A large (5 cm) ostectomy was performed in the right metatarsus of each animal. Each sheep was placed in dorsal recumbency on the surgical table. The right hind limb was clipped, prepped for aseptic surgery using alternating scrubs of povidone-iodine and alcohol, and draped. An interlocking nail (8 mm diameter, BioMedtrix, Whippany, NJ) was inserted retrograde from the distal metatarsus through a 2 cm skin incision made midway between the right and left fetlock condyles. After reaming, the nail was advanced to the distal third of the metatarsus. A 10 cm skin incision was then made through skin and subcutaneous tissues down to the mid-diaphyseal periosteum of the metatarsus. The overlying periosteum was elevated to expose the sub-periosteal cortical bone. The mid-diaphyseal ostectomy of 5 cm was then created using an oscillating saw with constant lavage for tissue cooling. In one group of two animals, a collagen sponge (Infuse Bone Graft, Medtronic, Minneapolis, Minnesota) soaked with rhBMP-2 (5.4 mg, 0.4 mg/mL) with no scaffold was placed in the ostectomy site (Fig. 6). In the other group of two animals, the 5 cm scaffold along with a collagen sponge soaked with rhBMP-2 (5.4 mg, 0.4 mg/mL) was placed in the ostectomy site. Before implantation, the collagen sponge was first inserted into the side hooks on the scaffold outer surface followed by injection of the 0.4 mg/mL rhBMP-2 solution to protect the sponge from compressive forces during or after implantation. The interlocking nail was then advanced across the ostectomy through the central channel of the scaffold and into the proximal metatarsal segment. An aiming jig arm was connected to the distal end of the nail to guide insertion of 4 locking bolts through the metatarsal surface and the transverse cannulations within the interlocking nail 2 on each side of the ostectomy. The surgical field was then thoroughly lavaged for wound closure. All animals were humanely euthanized 24 weeks following surgery by intravenous overdose of pentobarbitone sodium (88 mg/kg) in accordance with the American Veterinary Medical Associated (AVMA) guidelines.

### Ex vivo assessment

#### Non-destructive four-point bending

Post-sacrificial biomechanical evaluations were performed on treated metatarsi. Whole metatarsal bones underwent non-destructive four-point bending testing. The tests were conducted on a servo-hydraulic material testing machine (MTS Systems Corporation, Eden Prairie, Minnesota) equipped with a custom four-point bending fixture. The outer loading points were spaced 110 mm apart, and the inner loading points were spaced 70 mm apart, such that the entire defect length was within the two inner loading points and thus was subjected to a constant bending moment. The MTS crosshead was lowered at a rate of 0.1 mm/s to a maximum load of 300 N (150 N force at each loading point, 3 Nm moment in the central region). This load was great enough for the specimens to exhibit linear material behaviour without inducing permanent damage to the specimen. Specimens were loaded for four cycles for preconditioning, with data analysed from the fifth cycle. The bending stiffness was calculated as the slope of the linear elastic region of the global force–displacement loading curve.

#### Destructive torsion tests

Following four-point bending tests, the treated metatarsi underwent destructive torsion testing. The proximal and distal ends of the metatarsal bones were potted in an epoxy resin, and the potting material was clamped in a custom fixture on a torsional servo-hydraulic material testing machine (MTS Systems Corporation). The specimens were loaded in torsion at a rate of 0.5 degrees/s (0.0087 radians/s) until failure. The torsional rigidity was calculated as the slope of the linear elastic region of the torque versus normalized rotational displacement curve, where the normalized rotational displacement is the rotational displacement divided by the tested length of the specimen measured between the potting material surfaces. The ultimate torque was calculated as the maximum torque before failure, and the yield torque was determined by creating a line parallel to the linear elastic region of the torque-displacement curve, offset by one degree of rotation (0.017 radians), and finding the intersection of this line with the experimental curve.

#### Micro-computed tomography

Micro-CT scans were conducted with 37 μm resolution on the experimental scaffolds prior to implantation to determine initial volume, porosity, and pore size (Scanco micro-CT 80, Scanco USA Inc., Wayne, Pennsylvania). Following biomechanical testing on the day of sacrifice, the metatarsal bone segments containing the entire 5 cm implanted region were placed in 10% neutral buffered formalin for storage and micro-CT scanning. Scans were conducted with 37 × 37 × 37 μm voxel size, 70 kV potential, 500 ms integration time, 114 μA intensity, and 7.98 W power. The scans of the scaffold treatment were quantitatively analyzed in the scaffold volume ROI, not including the intramedullary space, as well as the peripheral ROI surrounding the scaffold (Fig. 2). Since the control group contains no scaffold, and therefore no landmarks to define separate ROIs, the control specimen scans were analyzed in a single ROI containing the entire defect volume outside of the intramedullary space. Bone volume within the ROI was computed as the volume with a mineral density between 220 and 1000 mg/cm^3^ hydroxyapatite. Scaffold volume was defined using a lower threshold of 125 and an upper threshold of 200 mg/cm^3^ hydroxyapatite. Additionally, the percent of new bone was calculated as the total volume of new bone measured relative to the bone volume measured in a healthy ovine metatarsus in an equivalent 5 cm Sect. (5190 mm^3^ bone volume).

#### Histology, histomorphometry, and histopathology

Following complete fixation in 10% neutral buffered formalin, all specimens were processed for undecalcified histology. The tissue was embedded in Acrylosin hard resin (Dorn and Hart Microedge Inc., Loxley, Alabama), cut and ground in the sagittal plane to a thickness of 70 μm, and polished. Three slides were produced from each specimen, and each slide included the entire implant area and surrounding bone but did not include any fractured segments from mechanical testing. Slides were stained with Sanderson’s Rapid Bone stain to visualize cells and allow detection of cartilage within the tissue. A counterstain was then applied using Van Gieson bone stain to allow differentiation of collagen and detection of immature woven bone and mature lamellar bone.

Calibrated digital images were captured at 10× magnification. The images were analyzed using ImagePro Premier software (version 9.3, Media Cybernetics, Silver Spring, Maryland) to measure the percent area of bone, soft tissue, and scaffold within the ROI. The ROI was defined by the scaffold area for the scaffold specimens and as the entire defect area for the control specimens (Fig. 3). Additionally, the area of peripheral bone growth was calculated for the scaffold specimens (Table 3, Fig. 3). These values were calculated as the mean measurement from the three slides per specimen.

Stained histology sections were evaluated by a board-certified veterinary pathologist blinded to the treatment group. The sections were analyzed and semi-quantitatively scored for the presence of polymorphonuclear cells, lymphocytes, plasma cells, macrophages, giant cells, necrosis, osteoblast cells, osteoclast remodeling, neovascularization, fibrosis, signs of implant degradation, and particulate debris. These parameters were graded on a 0 to 4 scale where 0 indicated no presence or activity and 4 indicated the greatest presence or activity. A cumulative inflammation score was calculated for each specimen as the sum of the scores of the polymorphonuclear cell, lymphocyte, plasma cell, macrophages, and giant cell categories. Additionally, the osteogenic response was assessed by grading the proportion of mineralized bone filling the implant pores or defect space. Specimens were graded on a 0 to 4 scale where 0 indicated no filling, 1 indicated less than 25% of the pore space was filled, 2 indicated between 25 and 50% of the pore space was filled, 3 indicated greater than 50% of the pore space was filled, and 4 indicated complete filling of the implant pores.
